# Convergent and divergent mechanisms of sugar recognition across kingdoms

**DOI:** 10.1016/j.sbi.2014.07.003

**Published:** 2014-10

**Authors:** Maureen E Taylor, Kurt Drickamer

**Affiliations:** Department of Life Sciences, Imperial College, London SW7 2AZ, United Kingdom

## Abstract

Protein modules that bind specific oligosaccharides are found across all kingdoms of life from single-celled organisms to man. Different, overlapping and evolving designations for sugar-binding domains in proteins can sometimes obscure common features that often reflect convergent solutions to the problem of distinguishing sugars with closely similar structures and binding them with sufficient affinity to achieve biologically meaningful results. Structural and functional analysis has revealed striking parallels between protein domains with widely different structures and evolutionary histories that employ common solutions to the sugar recognition problem. Recent studies also demonstrate that domains descended from common ancestors through divergent evolution appear more widely across the kingdoms of life than had previously been recognized.

## Highlights

• Carbohydrate-recognition domains fall into multiple fold families.
• Many of these domains are in multidomain proteins that also have other activities.
• Convergent evolution has led to shared features in domains with different folds.
• Families found across the kingdoms of life reflect extensive divergent evolution.
• Polymorphisms that affect sugar binding reflect recent evolutionary pressure.

**Current Opinion in Structural Biology** 2014, **28**:14–22

This review comes from a themed issue on **Carbohydrate-protein interactions and glycosylation**

Edited by **Harry J Gilbert** and **Harry Brumer**

For a complete overview see the Issue and the Editorial

Available online 5th August 2014

**http://dx.doi.org/10.1016/j.sbi.2014.07.003**

0959-440X/© 2014 The Authors. Published by Elsevier Ltd. This is an open access article under the CC BY license (http://creativecommons.org/licenses/by/3.0/).

## Introduction

Proteins that seem to have a primary function of binding sugars are often referred to as lectins, a term used initially in the context of plant seed proteins and then broadened to include examples from a wider range of species [1]. However, the names of many proteins that have sugar-binding activity are based on other biological functions that they have. For example, plant toxins represent a group of proteins in which sugar-binding activity in one part of a protein is used to target killing functions of another part of the protein. Similarly, sugar-binding proteins in yeast are usually denoted by their functions in flocculation and in adhesion. Bacterial proteins that interact with oligosaccharide ligands include adhesins, on fimbriae and pili [2], as well as toxins, but there is also sugar-binding activity associated with many glycosidases that contain non-catalytic carbohydrate-binding modules [3]. It is also common to use alternative designations such as glycan-binding proteins or glycan-binding receptors, particularly in the case of animal lectins.

In spite of this diversity of names and functions, a common feature of all of these proteins is that the sugar recognition function in each protein is mediated by a discrete protein module. The term carbohydrate-recognition domain is often used as a general label that encompasses all of the diverse folds, functions and sites of expression. However, many of the individual groups described in this review have other, more common designations and no systematic revision of the nomenclature seems appropriate at this point. Nevertheless, it is important that the diversity of names and categories does not obscure many evolutionary relationships between carbohydrate-recognition proteins, domains and modules in different species and kingdoms of life arising through divergent evolution as well as interesting similarities in the mechanisms of carbohydrate recognition that have come about through convergent evolution.

## Carbohydrate recognition in multiple protein fold families

One approach to comparing mechanisms of sugar recognition is to classify carbohydrate-recognition domains based on their sequences and structures. Two key conclusions emerge from such comparisons and the resulting classifications. First, sugar-binding activity can appear in the context of many different protein folds. Second, the protein folds of carbohydrate-recognition domains are not exclusively associated with sugar-binding activity. The first of these conclusions reflects independent evolution of this activity on multiple occasions and means that there is no simple way to identify sugar-binding proteins by looking for one particular protein fold [4]. The second conclusion means that similarity in the fold of a novel domain to a fold that can support sugar-binding activity does not necessarily imply that the new domain will bind sugars.

The principle that fold does not necessarily imply function is well established in the case of the C-type carbohydrate-recognition domains of animal lectins, which are a subset of the broader family designated C-type lectin-like domains that includes many members that lack sugar-binding activity [5], including some for which reports of sugar-binding activity have recently been called into question [6]. In many cases, other target ligands, such as lipoproteins or other proteins, are known but in other instances the functions of these domains remain to be established. Similar principles are evident for mannose 6-phosphate receptor homology domains (MRH domains), only some of which bind sugars, while others have ligands such as insulin-like growth factor II [7]. The same ideas emerge again for Fbs proteins that target tagging of proteins with ubiquitin by binding to the chitobiose core of N-linked glycans [8]. The Fbs proteins are part of a larger family of F-box proteins, most of which do not bind sugars and in fact at least one Fbs protein appears to lack this activity [9].

## Convergence on shared features in monosaccharide-specific sites

**Figure 1:**
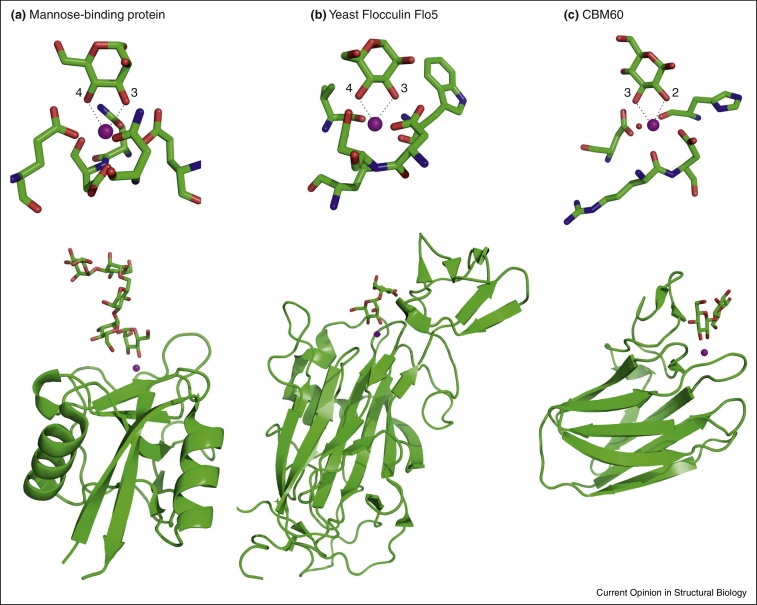
Involvement of Ca^2+^ in sugar-binding sites in the context of multiple different protein folds. Top panels show close-up views of sugar-binding sites and lower panels show overall folds of carbohydrate-recognition domains. **(a)** Human serum mannose-binding protein (2MSB) with Man_5_ oligosaccharide. **(b)** Yeast flocculin Flo5 (2XJS) with bound mannobiose. **(c)** Family 60 carbohydrate-binding module (CBM60) from *Cellvibrio japonicus* xylanase (2XFD) with bound cellobiose. Ca^2+^ is indicated as a magenta sphere in each panel and water is represented as a red sphere. Coordination bonds from adjacent equatorial hydroxyl groups to the Ca^2+^ are indicated as dashed lines.

A further consequence of these insights is that common features in the mechanisms of recognition of sugars that transcend fold families reflect convergent evolution. Two such features that crop up remarkably often are packing of sugars against aromatic residues and involvement of Ca^2+^. The former type of interaction, particularly between the apolar B face of galactose and a tryptophan residue, has been extensively discussed [10]. Ligation of sugars to Ca^2+^ ions was first described for the C-type carbohydrate-recognition domains in animal lectins [11], but has recently been identified in several other groups of sugar-binding proteins with carbohydrate-recognition domains from different fold families (Figure 1). Examples include yeast flocculation proteins [12] and adhesins [13^••^,14^••^] and at least two families of bacterial carbohydrate-binding modules [15], as well as the processing mannoside from the endoplasmic reticulum [16]. Other sugar-binding proteins that employ a pair of Ca^2+^ in sugar-binding sites are the pentraxin serum amyloid protein [17] and the lectin from *Pseudomonas aeruginosa* [18]. The convergent use of Ca^2+^ ligation in different structural contexts reflects the fundamental chemistry of the sugars, which are known to bind free Ca^2+^ [19].

In contrast to the cases noted above, Ca^2+^ and other divalent cations are sometimes indirectly involved in sugar binding, because they stabilize the sugar-binding conformation of a carbohydrate-recognition domain, for example in legume lectins [20], calnexin and calreticulin [21,22] and at least one family of bacterial carbohydrate-binding modules [23]. An interesting recent example of such an arrangement is seen in the mammalian L-type lectins ERGIC-53 and VIP36. On the basis of recent structural analysis of ERGIC-53, also known as LMAN1, it has been suggested that modulation of binding by different Ca^2+^ concentrations occurs in various luminal compartments in cells [24^••^]. This phenomenon appears to be a more subtle form of modulation of sugar-binding activity than that observed for the endocytic C-type lectins, in which loss of Ca^2+^ binding at endosomal pH results in loss of sugar binding activity, which provides a means of separating endocytic cargo from the receptors [25,26].

## A proliferation of secondary binding sites

A further interesting comparison of convergent sugar-binding sites is that, within fold families, there are often common mechanisms of binding to a core monosaccharide in a primary binding site, but diversity in binding of oligosaccharide and glycoconjugate ligands is achieved through extended and secondary binding sites that are unique to individual members of the family. Such extensions can involve interactions with additional sugar residues in an oligosaccharide ligand, but an increasing number of examples demonstrate binding to other modifications of the sugars.

Differences in secondary or extended binding sites often provide specificity for different oligosaccharides in closely related proteins. For example, the sorting lectins ERGIC-53 and VIP36 bind to distinct groups of high mannose oligosaccharides using a common primary mannose binding site that is extended in different ways. Because of these differences, ERGIC-53 binds to any Manα1-2Man disaccharides, including one bearing Glcα1-3 substitution on the non-reducing terminus [24^••^] compared to the selectivity of VIP36 for the unglucosylated Manα1-2Manα1-2Manα1-3 arm of high mannose oligosaccharides [27].

**Figure 2:**
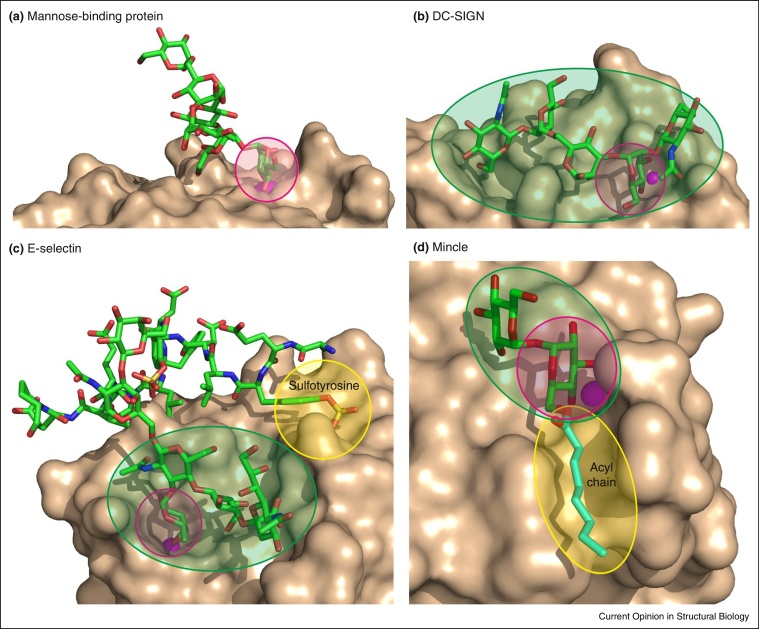
Multiple different ways in which binding specificity of C-type carbohydrate-recognition domains is enhanced by extended and accessory binding sites. Each of the binding sites involves a primary interaction between the Ca^2+^, shown in magenta, and two adjacent hydroxyl groups on a monosaccharide residue. **(a)** The relatively open binding site in mannose-binding protein binds only a terminal mannose residue, so only this residue interacts with the protein (2MSB). **(b)** DC-SIGN binds a more complex Man_3_GlcNAc_2_ oligosaccharide through an extended binding site that accommodates sugars on either side of the mannose residue in the primary binding site (1K9J). **(c)** In addition to ligation of fucose to Ca^2+^, the sialyl Lewis^x^ oligosaccharide interacts with an extended binding site in E-selectin, which also has an accessory binding site for sulfated tyrosine residues on a glycoprotein ligand (1G1S). **(d)** Mincle binds to the disaccharide trehalose as a result of one glucose residue binding in the Ca^2+^ site and the second glucose residue contacting an adjacent site. In addition, glycolipid binding is enhanced through an accessory site that forms a hydrophobic grove which can interact with acyl chains on the 6-OH groups of the glucose residues (4KZV). Primary binding sites are highlighted in pink, extended oligosaccharide-binding sites are indicated in green and accessory sites for other modifications are shaded yellow.

In the C-type lectin family, multiple examples of secondary interactions with sugars are common, leading to binding of high mannose and Lewis^x^ motifs, for example [28] and non-sugar substituents such as sulfate can also be accommodated in secondary binding sites [29]. In a novel twist, the macrophage receptor mincle has recently been shown to bind trehalose, the glucose α1-1 glucose disaccharide, through such a mechanism, but further specificity for mycobacterial glycolipids that bear this headgroup is achieved through interactions with the attached hydrophobic acyl chains, apparently through an adjacent hydrophobic groove [30^••^] (Figure 2).

In the case of the mannose 6-phosphate receptors, a mechanism involving a mannose-binding site extended by secondary interactions with a phosphate substituent is well established [31]. Elaboration of the secondary binding site, leading to selectivity for a GlcNAc residue attached to mannose through a phosphodiester linkage, can be achieved by a combination of removing potential hindrance to binding of the GlcNAc residues with addition of favourable secondary interactions between the protein and the added sugar [32]. The MRH domain in the OS-9 protein, which forms part of the quality control system of the endoplasmic reticulum, provides an alternative variation on the binding site in which a pair of tryptophan residues extends the primary mannose-binding site, making it selective for oligosaccharides containing Manα1-6Man units [33]. In contrast, recent analysis of the MRH domain in endoplasmic reticulum glucosidase II reveals an open binding site that lacks any of these extensions and thus represents a more prototypical mannose-binding site [34^••^].

## Common approaches to combining binding sites

In addition to convergence in the way that binding sites in individual domains work, the arrangement of these domains within proteins shows some interesting parallels between different groups of sugar-binding proteins. The phenomenon of enhanced binding to multivalent ligands through clustering of binding sites in oligomers is well established and has been extensively investigated for many types of sugar-binding proteins [35,36].

**Figure 3:**
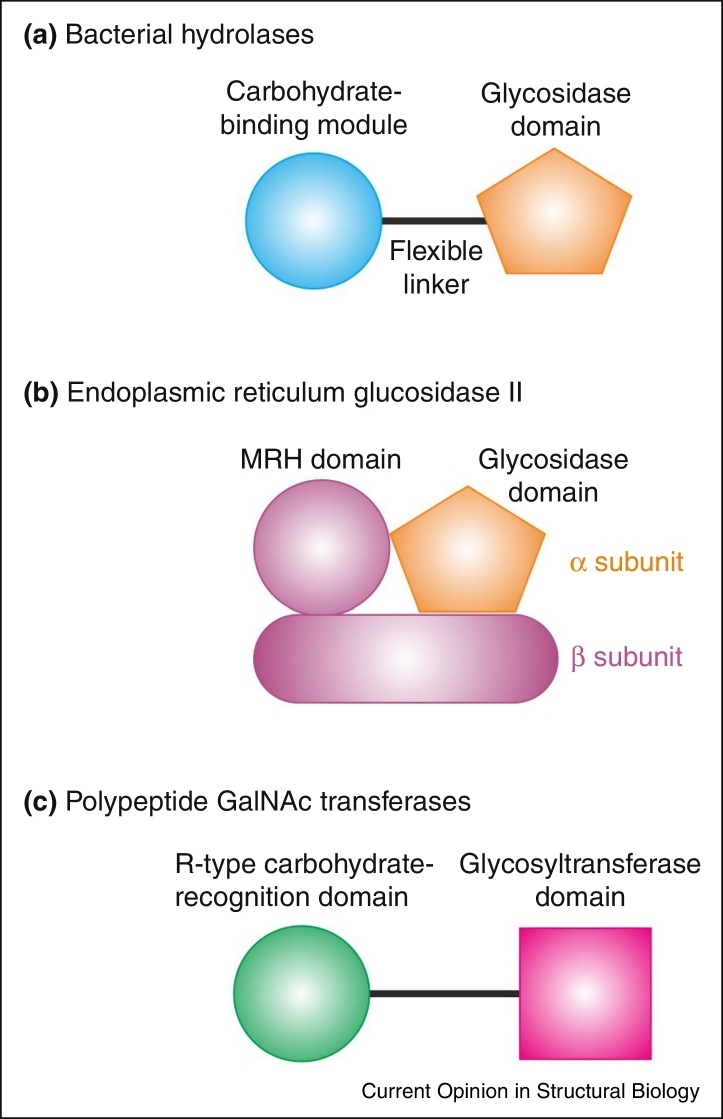
Association of carbohydrate-recognition domains with enzymatically active domains. **(a)** One or more carbohydrate-binding modules are often linked to bacterial glycosidases and cellulose-degrading enzymes in a single polypeptide. The carbohydrate-binding modules localize the activity on substrates and enhance the activity of enzymes. **(b)** The α subunit of endoplasmic reticulum glucosidase II contains the glucosidase active site, but the activity of the enzyme on high mannose oligosaccharides that bear terminal glucose residues on one branch is enhanced by the β subunit, which contains an MRH domain that binds mannose on another branch of the oligosaccharide. **(c)** R-type carbohydrate-recognition domains in many of the polypeptide GalNAc transferase proteins direct the enzyme to regions of substrate glycoproteins that already bear one or more GalNAc residues.

Somewhat less appreciated has been the generality of an arrangement in which a carbohydrate-recognition domain targets and enhances the activity of an enzyme that builds or degrades carbohydrates (Figure 3). The most extensively studied examples of such an arrangement are the carbohydrate-binding modules linked to the catalytic domains of many polysaccharide hydrolases [37^•^]. The recent demonstration of how an MRH domain linked to the catalytic domain of endoplasmic reticulum glucosidase II enhances the activity of this enzyme on nascent N-linked glycans demonstrates that similar pairings of sugar-binding and catalytic domains can be achieved using completely different structural elements [34^••^]. The same principle is seen in the large family of polypeptide *N*-acetylgalactosaminyltransferases, but in these cases it is R-type carbohydrate recognition domains coupled to synthetic enzymes that target the enzymes to sites adjacent to already glycosylated residues [38,39^•^]. Recent examples also illustrate how a carbohydrate-binding module can effectively extend the active site of a hydrolase [40] and that PA14 carbohydrate-binding modules can be inserted into the hydrolase domains rather than just being appended to them [41,42].

## Some old distinctions becoming less clear

It is increasingly difficult to delineate well defined subgroups of sugar-binding proteins based on any features other than sequence similarity. For example, as noted in the previous section, a domain organization linking a domain that recognizes sugars with one that catalyses modification of the sugar is no longer just a feature of the carbohydrate-binding module/glycosidase family. At the same time, some of the carbohydrate-binding modules linked to hydrolase domains are structurally related to lectins that are separate from catalytic domains [43]. Thus, a particular domain organization is not uniquely associated with a particular structural group of carbohydrate-recognition domains. Similarly, while hydrolase-associated carbohydrate-binding modules are often associated with binding of internal sugars in polysaccharide chains, a significant subgroup of these domains are now known to bind non-reducing terminal residues [37^•^]. Conversely, not all proteins referred to as lectins bind terminal residues, since the ability of galectins in interact with residues within a polypeptide is now well established [44].

**Figure 4:**
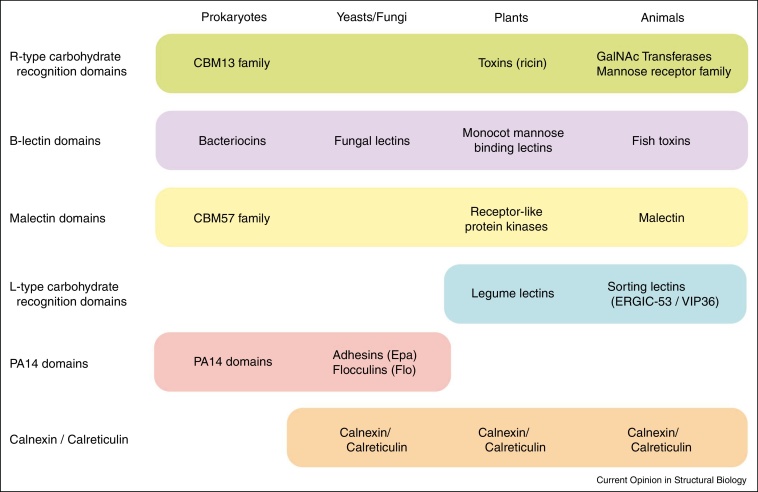
A summary of some of the types of carbohydrate-recognition domains that are found in a wide range of species. Other types of domain not shown are expressed only in more restricted groups of organisms. For example, galectins, siglecs and C-type lectins are expressed only in animals and several classes of adhesins are specific to bacteria.

Perhaps the most interesting recent change in the perception of different groups of carbohydrate-recognition domains is the finding that many of the families, for which sequence similarity provides strong evidence of divergence from a common ancestor, appear in a more diverse range of species and even kingdoms of life than was previously appreciated (Figure 4). It was previously recognized that structurally related domains used for different functions appear across the animal and plant kingdoms, since L-type carbohydrate-recognition domains are found in the legume lectins in plants as well as the sorting lectins such as ERGIC-53 and VIP36 in animal cells [24^••^,27]. It is now clear that structurally related carbohydrate-binding domains are present in both eukaryotes and prokaryotes. One of the most widely spread type of domain is the R-type carbohydrate-recognition domain, originally described in plant toxins such as ricin [45] and more recently recognized in polypeptide N-acetylgalactosaminyltransferases [39^•^] and the mannose-receptor family of proteins in animals [46] as well as the bacterial glycoside hydrolases containing CBM13 modules [47]. A second widely represented family of domains is the monocot mannose B-lectin type domain, widely studied in plants but also described in fish and fungi [48,49] and more recently in bacteria, including bacteriocins from *Pseudomonas* [50^••^,51^••^]. A third family that spans from prokaryotes to eukaryotes is the PA14 domain, which exhibits carbohydrate binding activity both as a carbohydrate-binding module in bacterial glycosidases and in yeast adhesions and flocculation factors [52]. A further unexpected sequence relationship is that between the endoplasmic reticulum sorting lectin malectin [53] and the CBM57 family of carbohydrate-binding modules of bacterial glycosidases and similar domains in putative plant kinases [54^•^], although in the latter case the apparent sequence similarities remain to be followed up with evidence for structural similarity and sugar-binding activity. These observations reflect the role of divergence of sugar binding domains as well as importance of convergence on similar recognition principles.

## Polymorphism analysis

A number of interesting patterns have been observed in the evolution of several of the families of glycan-binding receptors. Within the mammalian families, some types of receptors, such as those involved in intracellular trafficking of glycoproteins, are often relatively conserved across species, but some of the cell surface receptors tend to be more divergent. Extreme examples of such divergence include the DC-SIGN homologs [55] and the CD33-related siglecs [56]. Evolution of variability in receptors of the innate immune system probably reflects selective pressure from rapidly changing pathogens, many of which can exploit glycan-binding receptors as a means of entering cells.

In addition to variation between species, selection pressure from pathogens has led to establishment of polymorphisms in some of the receptors. The best studied example of a balanced polymorphism is in serum mannose-binding protein, in which several variants that have reduced capacity to activate complement have been identified [57]. The structural basis for how changes in the structure of the collagen-like domains in mannose-binding protein affect the interaction with complement is at least partially understood [58]. There is strong genetic evidence that other polymorphisms that result in amino acid substitutions in glycan-binding receptors of the innate immune system can affect susceptibility to infection. For example, polymorphisms in the C-type carbohydrate-recognition domains of the mannose receptor are linked to susceptibility to leprosy [59] and variability in the number of repeats in the neck region of DC-SIGNR, the endothelial paralog of DC-SIGN, may impact on transmission of human immunodeficiency virus [60]. However, in these latter cases, the molecular mechanisms that underlie the phenotypic consequences of changes to the amino acids sequences of these proteins remain to be established.

Other variations in the sequences of glycan-binding receptors have been more directly linked to changes in the sugar-binding activity of these proteins. Recent studies on langerin reveal that a form of the protein containing two amino acid changes compared to the most common reference form undergoes a major change in ligand binding, in which the ability to bind glycans terminated with galactose 6-sulphate is lost, while the affinity for glycans terminating in *N*-acetylglucosamine is increased [61^••^]. In this case, the amino acid changes are directly in the binding site and a structural basis for the changes in sugar binding has been demonstrated. The langerin results provide a paradigm for a novel way in which the diversity of glycan-binding receptors can be increased. In contrast, although there is increasing genetic evidence for linkage of risk of coronary artery disease with variants in the epidermal growth factor domain adjacent to the C-type carbohydrate-recognition domain of E-selectin [62], recent attempts to verify previous suggestions that these changes alter the sugar-binding activity of the receptor have been unsuccessful [63^•^]. From a structural perspective, this outcome is probably not surprising, given that the polymorphism is distant from the ligand-binding site and in a separate domain.

## Conclusions

It is clear that there is no single set of unifying principles that describe carbohydrate recognition across all the kingdoms of life. Nevertheless, the examples described in this short review illustrate that some of the solutions to the sugar recognition problem go back very far in evolution and that mechanisms for binding sugars based on the chemical properties of the sugar ligands can be implemented in the context of many different protein folds. The first of these conclusions provides a useful basis for identifying potential sugar recognition systems from genomic sequence data. However, the second point means that novel carbohydrate-recognition domains which utilize different protein scaffolds may still remain to be discovered.

## References and recommended reading

Papers of particular interest, published within the period of review, have been highlighted as:

• of special interest
•• of outstanding interest
